# Adjuvant EGFR-TKIs for Patients With Resected EGFR-Mutant Non-Small Cell Lung Cancer: A Meta-Analysis of 1,283 Patients

**DOI:** 10.3389/fonc.2021.629394

**Published:** 2021-04-12

**Authors:** Rui-Lian Chen, Ling-Ling Sun, Yang Cao, Han-Rui Chen, Jing-Xu Zhou, Chu-Ying Gu, Ying Zhang, Si-Yu Wang, Wei Hou, Li-Zhu Lin

**Affiliations:** ^1^ Integrative Cancer Centre, The First Affiliated Hospital of Guangzhou University of Chinese Medicine, Guangzhou, China; ^2^ Department of Oncology, Guang’anmen Hospital, China Academy of Chinese Medical Sciences, Beijing, China; ^3^ Cancer Project Team of China Center for Evidence Based Traditional Chinese Medicine, Beijing, China; ^4^ Department of Thoracic Surgery, Sun Yat-sen University Cancer, Guangzhou, China

**Keywords:** adjuvant EGFR-TKIs, non-small-cell lung cancer, EGFR mutation, resected, meta-analysis, brain recurrence

## Abstract

**Background:**

Cisplatin-based chemotherapy was previously considered as the standard adjuvant therapy for improved overall survival (OS) in patients with non-small cell lung cancer (NSCLC) after surgery. However, the benefit was limited due to high risks of recurrence and adverse events. In the present study, the efficacy of adjuvant epidermal growth factor receptor tyrosine kinase inhibitors (EGFR-TKIs) for EGFR-mutant patients after surgery was investigated using the latest updated data.

**Methods:**

This meta-analysis included a comprehensive range of relevant studies identified from database searches. Disease-free survival (DFS) and OS with hazard ratios (HRs) were calculated using random-effect or fixed-effect models. Subgroup analysis was also performed.

**Results:**

A total of seven randomized clinical trials were included in the meta-analysis and involved 1,283 NSCLC patients harboring EGFR mutations. In resected EGFR-mutant NSCLC patients, adjuvant EGFR-TKIs were significantly better than chemotherapy in terms of DFS (HR: 0.41; 95%CI: 0.24–0.70, P = 0.001), without showing any benefit in OS (HR: 0.72; 95%CI: 0.37–1.41, P = 0.336). No significant difference in DFS was observed between patients with EGFR exon 19 deletion and those with L858R mutation. Resected EGFR-mutant NSCLC patients treated with osimertinib experienced improved DFS and a lower risk of brain recurrence than those treated with gefitinib or erlotinib. Adjuvant EGFR-TKIs reduced the risk of bone and lung relapse, without decreasing the risk of local recurrence and liver relapse.

**Conclusion:**

This meta-analysis shows that adjuvant EGFR-TKI therapy could significantly prolong DFS in patients with resected EGFR-mutant NSCLC. Treatment with osimertinib showed improved DFS with a lower risk of brain recurrence than treatment with gefitinib or erlotinib for resected disease.

## Introduction

Lung cancer remains the leading cause of cancer-related mortality worldwide (1). Among the patients diagnosed each year with non-small cell lung cancer (NSCLC), 20–25% of cases with early-stage (I–IIIA) disease are suitable for surgical resection with curative intent (2). Postoperative cisplatin-based chemotherapy has been recommended as adjuvant treatment in resected NSCLC patients, except for subjects with stage IA and part of stage IB (3, 4). However, the therapy only resulted in a 16% decrease in the risk of disease recurrence and a 5% increase in 5-year overall survival (OS) (5).

Molecular-targeted drugs have been successfully used as adjuvant therapy for several types of cancers, for example, imatinib for gastrointestinal stromal tumors (6, 7). Epidermal growth factor receptor (EGFR) mutations are common oncogenic driver mutations in NSCLC patients, such as EGFR exon 19 deletion and L858R mutation. EGFR tyrosine kinase inhibitors (EGFR-TKIs) are considered as standard first-line treatment for advanced NSCLC harboring EGFR mutations, with improved progression-free survival (PFS) and quality of life (8, 9). This has promoted the investigation of their use as adjuvant therapy in resected patients.

The ADAURA trial showed that the disease-free survival (DFS) of patients treated with osimertinib was significantly longer than that with a placebo in resected EGFR-mutant NSCLC patients with stage IB to IIIA disease, consistent with the results of the CTONG 1104 and EVAN trials. However, in several previous trials, conflicting results regarding the efficacy of adjuvant EGFR-TKIs in resected NSCLC patients were reported (10–13). Furthermore, advanced NSCLC patients harboring EGFR exon 19 deletion experienced a better prognosis, compared with those harboring L858R mutation when treated with EGFR-TKIs (14, 15). In early-stage NSCLC disease, the difference in the efficacy of EGFR-TKI based on EGFR mutation status was not previously reported. In addition, the recurrence rate of resected NSCLC is approximately 30**–**75%, with poor postoperative morbidity (16, 17). Understanding the recurrence patterns after treatment with adjuvant EGFR-TKIs can help immediately identify the sites prone to recurrence, and the prognosis can be improved with appropriate surveillance strategies in clinical practice. However, evidence regarding the long-term tumor recurrence patterns after EGFR-TKIs as adjuvant therapy is scare. In advanced EGFR-mutant NSCLC disease, first-line treatment with osimertinib showed improved clinical benefit and reduced the risk of the central nervous system recurrence compared with gefitinib or erlotinib treatment (18). In resected disease, the difference in clinical outcome and brain relapse between treatment with osimertinib *versus* gefitinib or erlotinib has not been investigated. Thus, a meta-analysis to investigate the effects of adjuvant EGFR-TKIs is urgently needed. In the present study, the difference in clinical outcome based on the generation of EGFR-TKI or mutation status was investigated, and tumor recurrence patterns were explored with the subgroup analyses. The results of this study may provide more information and guidance for researchers and clinicians in the management of adjuvant therapy in resected EGFR-mutant NSCLC patients.

## Methods

### Study Eligibility and Selection

The PubMed, Embase, and the Cochrane Library databases were used in a systematic search for studies published up to September 20, 2020 with no start date limit applied. The search terms used were “lung cancer”, “adjuvant or resected or operable”, “erlotinib or gefitinib or icotinib or afatinib or dacomitinib or osimertinib” and “randomized control trial”. We also searched meeting abstracts from the American Society of Clinical Oncology, European Society for Medical Oncology, World Conference on Lung Cancer and American Association for Cancer Research for Medical Oncology websites.

Eligible studies that met the following criteria were included: Phase II or III randomized control trials (RCTs); and comparisons of survival in stage I–IIIA NSCLC patients treated with adjuvant EGFR-TKIs *versus* adjuvant chemotherapy or placebo; and studies with reported hazard ratios (HRs) for survival analysis (DFS or OS) or the number of events for disease relapse patterns and adverse events (AEs) in EGFR-mutant lung cancer from the overall patient population or subgroups analyses. The exclusion criteria were as follows: studies with irretrievable or insufficient data for statistical analysis; single-arm trials, observational studies, editorials, reviews, and commentaries; duplicate studies; and abstracts and studies written in languages other than English. Two authors (L-LS and H-RC) independently searched the databases and screened articles using the titles and abstracts to find potentially relevant studies.

### Data Extraction

All candidate articles were independently evaluated and extracted by two investigators (R-LC and J-XZ), and all discrepancies were resolved by the consensus among all authors. From each study, the first author name, clinical trial name, trial phase, EGFR mutation status, generation of adjuvant EGFR-TKIs, other baseline clinicopathologic characteristics, planned and received treatment and toxicity, survival outcomes, and relapse patterns were extracted. The quality of the included studies was independently assessed by two authors (YC and YZ), according to the five-point Jadad scoring system (19).

### Statistical Analysis

The HRs and 95% confidence intervals (CIs) for the DFS and OS of resected EGFR-mutant NSCLC patients were derived from the overall patient population and subgroups within each individual study. For dichotomous outcomes, the number of patients was used to calculate odds ratio (OR) estimates of trials with the Mantel–Haenszel method, such as disease relapse patterns and AEs.

There are two common statistical models for meta-analysis, the fixed-effect model and the random-effect model. The fixed-effect model depends on the hypothesis that all studies in the meta-analysis share a true effect size. In contrast, in the random-effect model, the true effect size may differ from study to study. The random-effect model is often considered as the appropriate model (20). Heterogeneity among the trials was assessed using the Q-test and was quantified with I^2^ values (21). An I^2^ statistic >50% or P value <0.05 was defined as significant heterogeneity among trials. If significant heterogeneity was observed, the random-effect model was used for analysis. If significant heterogeneity was not found, the fixed-effect model was applied (22). In addition, the funnel plot and the Begg’s and Egger’s tests were performed. All reported P-values were two sided, and the statistically significant level was set at 0.05. The meta-analysis was performed in accordance with recommendations from the Cochrane Collaboration and the Preferred Reporting Items for Systematic Reviews and Meta-Analyses guidelines, using Stata/SE version 16.0 software (Stata Corporation, College Station, TX, USA).

### Subgroup Analysis

A series of subgroup analyses were conducted to explore the effects of variables on the efficacy of EGFR inhibitors for resected EGFR-mutant NSCLC. The subgroup included EGFR mutation status (exon 19 deletion *vs.* L858R mutation), age (age ≥65 years *vs.* <65 years), sex (male *vs.* female), smoking status (smokers *vs.* non-smokers), histology (adenocarcinoma *vs.* non-adenocarcinoma), generation of EGFR-TKIs (gefitinib or erlotinib *vs.* osimertinib), and the relapse patterns.

## Results

### Characteristics of the Included Studies

**Figure 1:**
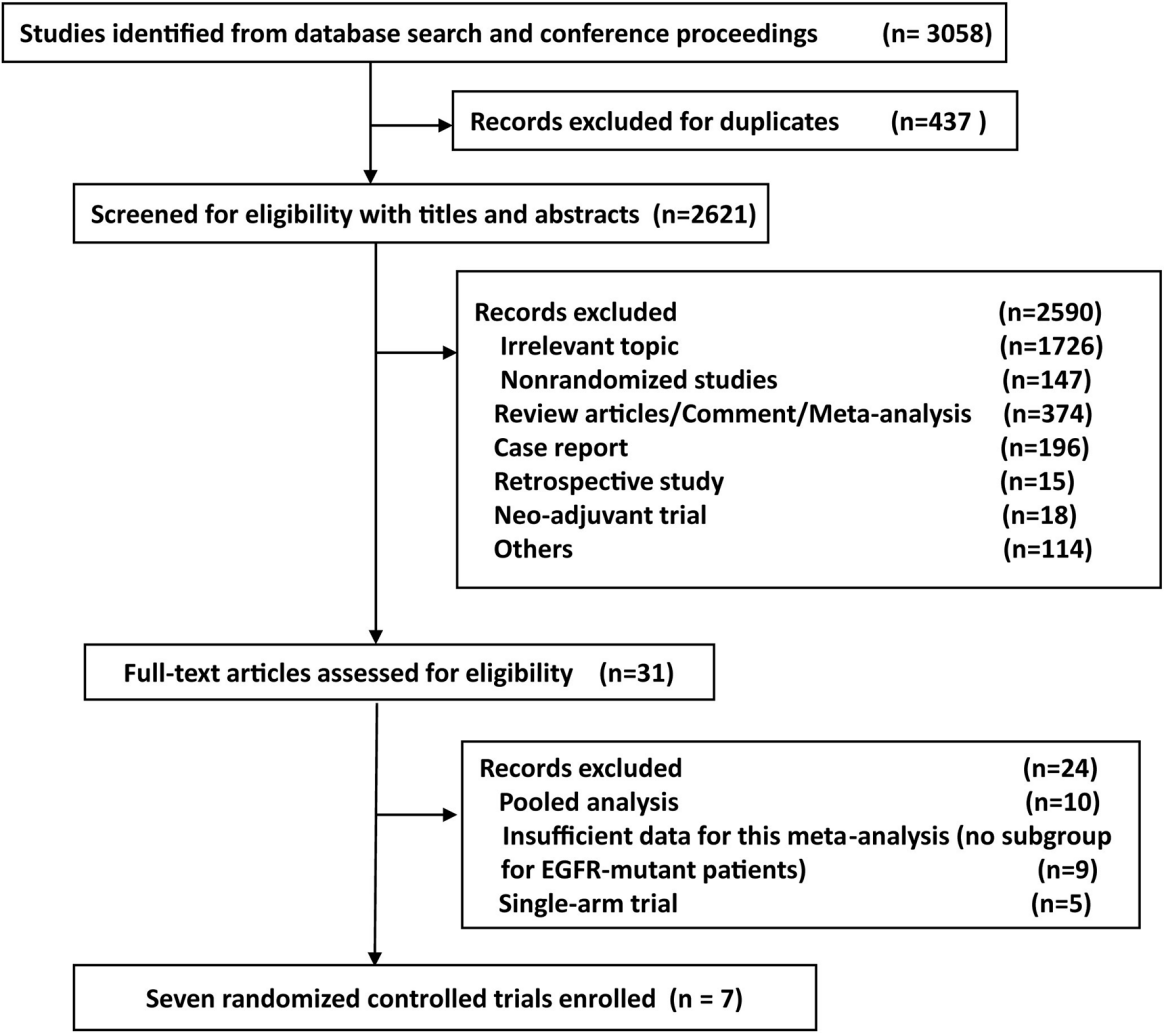
The flowchart of study selection process for the meta-analysis.

A total of 3,058 relevant records were identified from databases and conferences using our search strategy. After screening the titles and abstracts of the articles, the full texts of 31 articles were reviewed for eligibility (**Figure 1**). Among these, seven RCTs were finally considered eligible for our meta-analysis based on the inclusion and exclusion criteria. Detailed data on disease relapse in the CTONG 1104 trial was reported in another article by Xu et al. (23). A total of 1,283 EGFR-mutant patients were identified in seven studies (10–13, 24–26). All cases in five studies were diagnosed NSCLC with an activating EGFR mutation (12, 13, 24–26). The proportion of resected EGFR-mutant NSCLC patients was 16.5 and 3.0% in the RADIANT and NCIC CTG BR19 (CTSUBR19) studies, respectively (10, 11). The clinical characteristics and the quality assessment of the included studies are presented in **Table 1**.

**Table 1:** Characteristics of the included trials in the meta-analysis.

Clinical trials	Authors	Phase	Age (median)	Stage	Treatment groups	EGFR-mutant Patients	Disease-free survival	Overall survival	Quality assessment
							HR (95%CI)	HR (95%CI)	
**RADIANT** (11)	Kelly et al. (2015)	3	61	IB–IIIA	Erlotinib *vs* Placebo	161	0.61 (0.38–0.98)	1.09 (0.55–2.16)	6
**CTSUBR19** (10)	Goss et al. (2013)	3	NA	IB–IIIA	Gefitinib *vs* Placebo	15	1.84 (0.44–7.73)	0.83 (0.54–1.26)	5
**Li et al.** (24)	Li et al. (2014)	2	59.5	IIIA	Chemotherapy + gefitinib *vs* chemotherapy	60	0.37 (0.16–0.85)	0.61 (0.42–0.87)	3
**Feng et al.** (25)	Feng et al. (2015)	2	57	IB–IIIA	Chemotherapy + icotinib *vs* chemotherapy	41	0·22 (0·04–1.15)	0·86 (0·62–1·20)	3
**CTONG 1104** (13)	Zhong et al. (2018)	3	58	II–IIIA	Gefitinib *vs* VP	222	0.60 (0.42–0.87)	0.92 (0.62–1.36)	5
**EVAN** (12)	Yue et al. (2018)	2	59	IIIA	Erlotinib *vs* VP	102	0.27(0.14–0.53)	0.17(0.05–0.58)	5
**ADAURA** (26)	Wu et al. (2020)	3	NA	IB–IIIA	Osimertinib *vs* Placebo	682	0.20(0.15–0.27)	NA	6

VP, Vinorelbine plus cisplatin; NA, not available.

### Effects of EGFR-TKIs on DFS and OS in Patients With Resected EGFR-Mutant NSCLC

**Figure 2:**
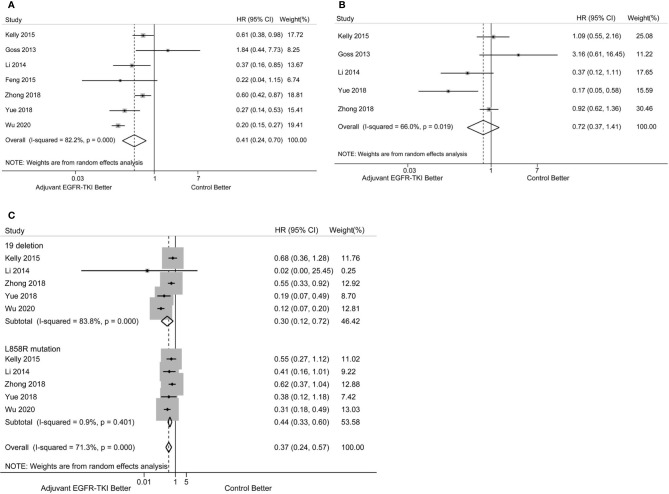
**(A)** Forest plot of hazard ratio (HR) for disease-free survival (DFS) with adjuvant EGFR-TKI *versus* the control group in EGFR-mutant resected NSCLC patients. **(B)** Forest plot of HR for overall survival (OS) with adjuvant EGFR-TKI *versus* control arms in EGFR-mutant resected NSCLC patients. **(C)** Forest plot of HR for DFS with adjuvant EGFR-TKI *versus* the control group in NSCLC patients with EGFR exon 19 deletion and L858R mutation.

EGFR-mutant NSCLC patients showed improved DFS after treatment with adjuvant EGFR-TKIs compared with the control group. Pooled HRs based on the seven RCTs indicated a lower risk of disease progression with EGFR-TKIs when compared with the control group (HR: 0.41; 95%CI: 0.24–0.70, P = 0.001, **Figure 2A**). Significant heterogeneity in DFS was observed among the trials (I^2^ = 82.2%, P < 0.001).

OS data were not available in the study by Feng et al. and not immature in the ADAURA trial. Thus, the analysis of OS was from five RCTs with available data. No significant improvement was observed between adjuvant EGFR-TKI therapy and the control group in resected EGFR-mutant NSCLC (HR: 0.72; 95%CI: 0.37–1.41, P = 0.336, **Figure 2B**). Significant heterogeneity in OS was found (I^2^ = 66.0%, P = 0.019).

### Effects of EGFR Mutation Status on DFS

To further explore the effects of EGFR mutation status on DFS, subgroup analyses of DFS in patients with exon 19 deletion *versus* L858R mutation were performed. The pooled survival estimates were based on 660 NSCLC patients harboring exon 19 deletion from seven RCTs and showed that EGFR-TKI treatment had a favorable effect on DFS (HR: 0.30; 95%CI: 0.12–0.72; P = 0.007, **Figure 2C**) There was significant heterogeneity in the analysis (I^2^ = 83.8%, P < 0.001). A total of 565 patients harboring L858R mutation experienced improved DFS with EGFR-TKI therapy compared with the control group (HR: 0.44; 95%CI: 0.33–0.60; P < 0.001, **Figure 2C**), with no significant heterogeneity (I^2^ = 0.9%, P = 0.401). No significant difference in DFS was observed between EGFR exon 19 deletion and L858R mutation subgroups (P _for heterogeneity_ = 0.290).

### Subgroup Analysis Based on Clinical Characteristics

**Figure 3:**
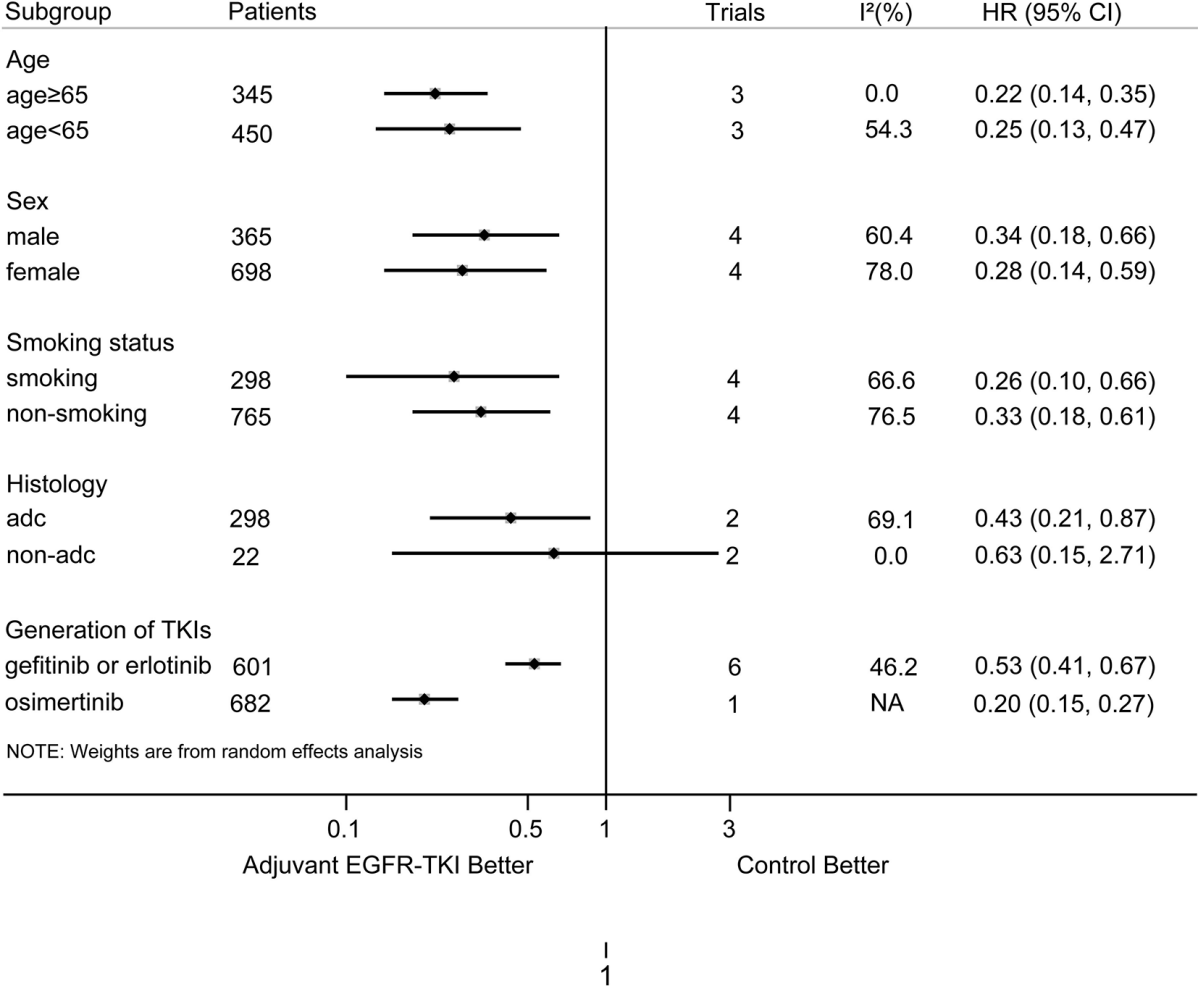
Subgroup analysis on DFS according to age, sex, smoking status, histology, and generation of EGFR-TKIs. NA, not available; adc, adenocarcinoma; non-adc, non-adenocarcinoma.

The results of our subgroup analyses are shown in **Figure 3**. For most of the subgroups (sex, age, smoking history, and generation of EGFR-TKIs), the DFS benefit of EGFR-TKIs was greater than the control group. For the histology subgroup, a significant DFS advantage of EGFR-TKIs was observed in EGFR-mutant NSCLC patients with adenocarcinoma, but not in those with non-adenocarcinoma. Furthermore, in patients with resected EGFR-mutant NSCLC, the DFS for osimertinib was longer than that for first-generation EGFR-TKIs, with a statistically significant difference (osimertinib *vs.* gefitinib or erlotinib, HR: 0.20; 95%CI: 0.15**–**0.27 *vs*. 0.53; 0.41**–**0.67; P _for heterogeneity_ < 0.001).

### Effects of EGFR-TKIs on Disease Relapse

**Figure 4:**
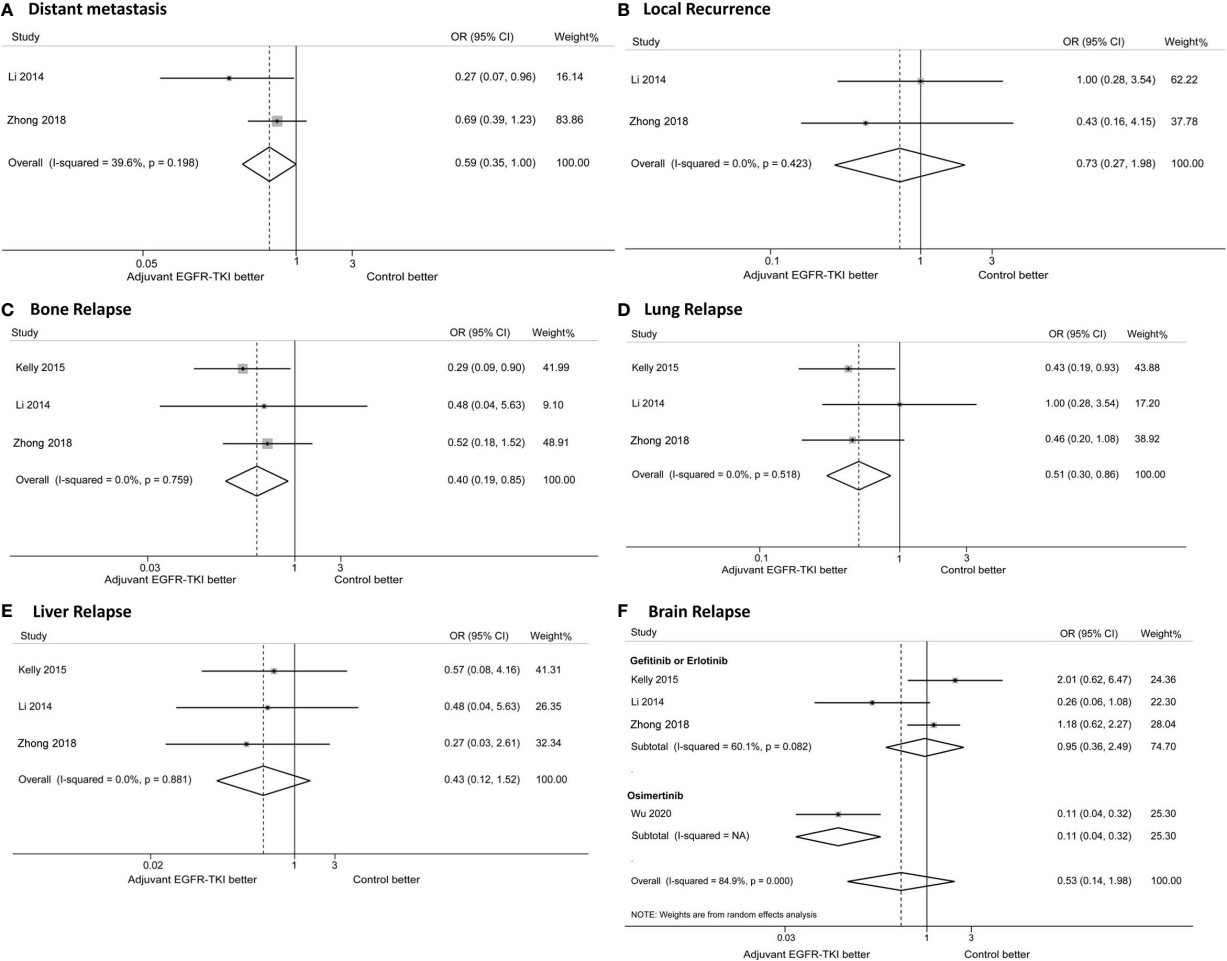
Forest plots of odd ratios (ORs) for distant metastasis **(A)**, local recurrence **(B)**, bone relapse **(C)**, lung relapse **(D)**, brain relapse **(D)**, liver relapse **(E)** and brain relapse **(F)**. * Due to no event was reported, the study by Li was excluded in the analysis of liver relapse, and the study by Kelly was excluded in the analysis of distant metastasis and local recurrence.

Data on disease relapse were not reported in the EVAN and RADIANT trials, or in Feng’s study. Based on the available data reported from four trials (the ADAURA trial only reported brain recurrence), the effects of EGFR-TKIs on disease relapse were analyzed. EGFR-TKI therapy reduced the risk of bone relapse (OR: 0.40; 95%CI: 0.19–0.85; **Figure 4C**) and lung relapse (OR: 0.51; 95%CI: 0.30–0.86; **Figure 4D**), without decreasing the risk of local recurrence (OR: 0.73; 95%CI: 0.27–1.98, **Figure 4B**) and liver relapse (OR: 0.43; 95%CI: 0.12–1.52, **Figure 4E**). The addition of EGFR-TKIs decreased the distant metastasis risk (OR: 0.59; 95%CI: 0.35–1.00; **Figure 4A**), although the difference was not statistically significant (P = 0.052).

No significant difference was observed in the brain recurrence with EGFR-TKIs compared with the control group (**Figure 4F**). Subgroup analysis was performed to evaluate the effects of different generations of EGFR-TKIs. The risk of brain relapse with osimertinib treatment was significantly lower than with gefitinib or erlotinib treatment (osimertinib, OR: 0.11; 95%CI: 0.04–0.32; gefitinib or erlotinib, OR: 0.95; 95%CI: 0.36–2.49; P _for heterogeneity_ < 0.001; **Figure 4F**). The incidence of brain recurrence was 1% (95%CI: 0–3%) in the osimertinib group and 17% (95%CI: 10–28%) in the gefitinib or erlotinib group, with a significant difference as shown in **Figure S1**.

### AEs

The AEs of EGFR-TKIs are shown in **Table 2**. Among the 623 EGFR-mutant patients treated with EGFR-TKIs, the rate of AEs of any grade was 86.92% (95%CI: 65.83–95.82%), and the rate of AEs of overall grade 3 or higher was 14.09% (95%CI: 8.23–23.07%). The most common severe AEs included rash (5.09%, 95%CI: 1.51–15.81%), diarrhea (2.57%, 95%CI: 1.58–4.15%), nausea or vomiting (1.93%, 95%CI: 1.10–3.36%), pneumonia (0.78%, 95%CI: 0.20–3.07%), and fatigue (0.35%, 95%CI: 0.05–2.44%).

**Table 2:** Severe Adverse events in EGFR-TKIs treatment arm.

Severe adverse events	Li et al. (24)	Kelly et al. (11)	Zhong et al. (13)	Yue et al. (12)	Wu et al. (26)	Incidence (95%CI),%
rash	2	19	1	2	NA	5.09 (1.51–15.81)
diarrhea	1	5	1	1	8	2.57(1.58–4.15)
nausea/vomiting	0	0	3	3	6	1.93(1.10–3.36)
Pneumonia	NA	0	1	1	NA	0.78 (0.20-3.07)
fatigue	0	1	0	0	NA	0.35 (0.05–2.44)
All ≥grade 3 AE	6	30	13	6	22	14.09 (8.23–23.07)
Any grade	28	93	61	29	327	86.92 (65.83–95.82)

AE, adverse event.

### Study Quality and Publication Bias

Randomized treatment allocation sequences were generated in all trials. Four trials were open-label, and three were double-blind. The Jadad score ranged from 3 to 6, indicating a high quality (**Table 1**). The funnel plot, as well as Egger’s and Begg’s tests, showed no publication bias in the overall or subgroup populations (all P > 0.05).

## Discussion

Our large meta-analysis of 1,283 patients with resected EGFR-mutant NSCLC from seven RCTs showed that patients treated with adjuvant EGFR-TKIs experienced improved DFS, with tolerated AEs, compared with chemotherapy or a placebo. No significant difference in OS was observed in the adjuvant EGFR-TKI group.

Conflicting results regarding the clinical benefit of adjuvant EGFR-TKIs in resected NSCLC patients were reported in previous trials. The CTSUBR19 (10) and RADIANT (11) and trials indicated that resected EGFR-mutant NSCLC patients did not obtain significantly improved clinical benefit of EGFR-TKI treatment; however, significant improved DFS with EGFR-TKIs was observed in the EVAN, CTONG 1104, and ADAURA trials (12, 13, 26). These conflicting results may be due to the different populations investigated in these studies. The EVAN trial and the study by Li et al. enrolled only patients with stage IIIA. All patients in the CTONG 1104 trial and 60% of patients in the ADAURA trial were stages II–IIIA (12, 13, 26). However, most patients in the CTSUBR19 and RADIANT trials were stages I–II (10, 11).

In some meta-analyses, NSCLC patients with EGFR mutation reportedly obtained clinical benefit from adjuvant EGFR-TKI therapy (27–30). However, those meta-analyses included retrospective studies, which are lower in quality than RCTs. Our study is the largest meta-analysis of resected EGFR-mutant NSCLC patients from RCTs to date. Compared with previous meta-analyses, the high quality of data strengthens the evidence on the efficacy of adjuvant EGFR-TKI therapy. In addition, the differences in clinical effects of EGFR-TKIs based on EGFR mutation status (exon 19 deletion *vs.* L858R mutation) were investigated in our meta-analysis. Furthermore, the effects of different generations of EGFR-TKIs and the long-term tumor recurrence patterns were analyzed. The heterogeneity of DFS and OS across studies was substantial in our meta-analysis, which may be due to sex, age, smoking history, histology, stage, and generation of EGFR-TKIs. In advanced NSCLC disease, patients harboring EGFR exon 19 deletion experienced improved PFS with EGFR-TKIs compared with those harboring L858R mutation (15, 31). However, in resected NSCLC, the difference in outcome between patients with these EGFR mutation types treated with adjuvant EGFR-TKIs was not investigated in any study. Our meta-analysis shows that the clinical benefit of adjuvant EGFR-TKIs was observed in early-stage patients harboring EGFR exon 19 deletion or L858R mutation; the difference between the two mutation types was non-significant. There are several possible complicated reasons why our result differs from those of advanced-stage patients reported in previous studies. First, the tumor burden in advanced NSCLC patients was higher than that in early-stage NSCLC cases after surgery. Therefore, the abundance of EGFR mutations may be high in patients with metastatic NSCLC. The clinical benefit of EGFR-TKIs was reportedly closely associated with the abundance of EGFR mutations (32). Second, the sample size of patients in the analysis of EGFR mutation status was not large in our meta-analysis; thus, additional studies with a larger cohort are necessary. The biological behavior of early-stage NSCLC may differ from that of advanced-stage NSCLC.

Our meta-analysis shows that resected EGFR-mutant NSCLC patients treated with osimertinib experienced significantly longer DFS and a reduced risk of brain recurrence, compared with those who received gefitinib or erlotinib. In preclinical studies, osimertinib could induce apoptosis and exert better effects against EGFR-mutant tumor compared with first-generation EGFR-TKIs, with significant results observed in xenograft and transgenic models (33, 34). In previous studies, osimertinib had better exposure in the brain than other EGFR-TKIs due to the greater penetration of the blood–brain barrier (35, 36). In addition, first-line treatment with osimertinib showed significant clinical benefit in terms of PFS and OS in advanced EGFR-mutant NSCLC patients with a decreased the risk of brain progression compared with gefitinib or erlotinib (18, 37, 38). Similar to EGFR-mutant patients with metastatic disease, the superior efficacy of osimertinib was also observed in those with resected NSCLC in our meta-analysis.

Currently, concerns regarding EGFR-TKIs as adjuvant treatment in resected NSCLC remain. First, the early use of EGFR-TKIs could change the biological behaviors in NSCLC patients and lead to more complicated resistance mechanisms, compared with those just waiting until disease recurrence. Treatment duration is another concern. EGFR-mutant patients enrolled in the CTONG 1104 and EVAN trials were treated with adjuvant gefitinib or erlotinib for two years or until disease recurrence; however, patients in the ADAURA trial were treated with adjuvant osimertinib for three years. Re-biopsy is widely used to evaluate the resistance mechanisms after disease relapse and to modify the treatment strategy accordingly. The biological behaviors of relapse disease after treatment with adjuvant EGFR-TKIs requires further investigation. Circulating tumor DNA (ctDNA) has been considered as an excellent predictor of disease recurrence and used to identify the molecular residual disease. In a study of patients with localized lung cancer treated with curative intent, ctDNA was detected in 94% of patients with disease relapse before radiographic recurrence at a median of 5.2 months post-treatment (39). Additional research is needed on using ctDNA to identify molecular residual disease to determine the EGFR-TKI treatment duration and provide personalized adjuvant treatment.

The present study has several limitations. First, data for the analysis of NSCLC patients was derived from published clinical trials rather than from each individual patient. Therefore, analyzing the influence of disease stage accurately is difficult. Second, part of the data in our meta-analysis were derived from subgroup analyses of published RCTs. Thus, some important information was not collected from subgroup results, such as smoking history, stage, sex, and EGFR mutation status. Due to the lack of information on the stage of each patient, specific subgroup analyses of stage I, stage II, and stage III patients could not be performed.

## Conclusion

Despite the above limitations, the results of the current study have significant implications. This meta-analysis indicates that adjuvant EGFR-TKI therapy brought significant clinical benefit in terms of DFS in resected EGFR-mutant NSCLC patients. Osimertinib had longer DFS with lower risk of brain recurrence than gefitinib or erlotinib for resected NSCLC; however, additional studies are warranted.

## Data Availability Statement

The original contributions presented in the study are included in the article/**Supplementary Material**. Further inquiries can be directed to the corresponding author.

## Author Contributions

Conception and design: L-ZL, WH, and R-LC. Data collection or management: R-LC, L-LS, H-RC and J-XZ. Data evaluation: YC and ZY. Statistical analysis: R-LC, L-LS and C-YG. Manuscript writing and revising: R-LC, S-YW and L-ZL. All authors contributed to the article and approved the submitted version.

## Funding

This study was supported by grant from Traditional Chinese medicine evidence-based capacity building project (grant number 2019XZZX–ZL001), Pilot project of Integrated traditional Chinese and western medicine clinical collaboration for major and difficult diseases (lung cancer).

## Conflict of Interest

The authors declare that the research was conducted in the absence of any commercial or financial relationships that could be construed as a potential conflict of interest.

## Supplementary Material

Supplementary Figure 1 Forest plots ot the incidence of brain recurrence.
