# Supplementary figures and images for: Adjuvant EGFR-TKIs for Patients With Resected EGFR-Mutant Non-Small Cell Lung Cancer: A Meta-Analysis of 1,283 Patients
Source: Front Oncol. 2021 Apr 12;11:629394. doi: 10.3389/fonc.2021.629394 (PMC8071858; doi:10.3389/fonc.2021.629394)

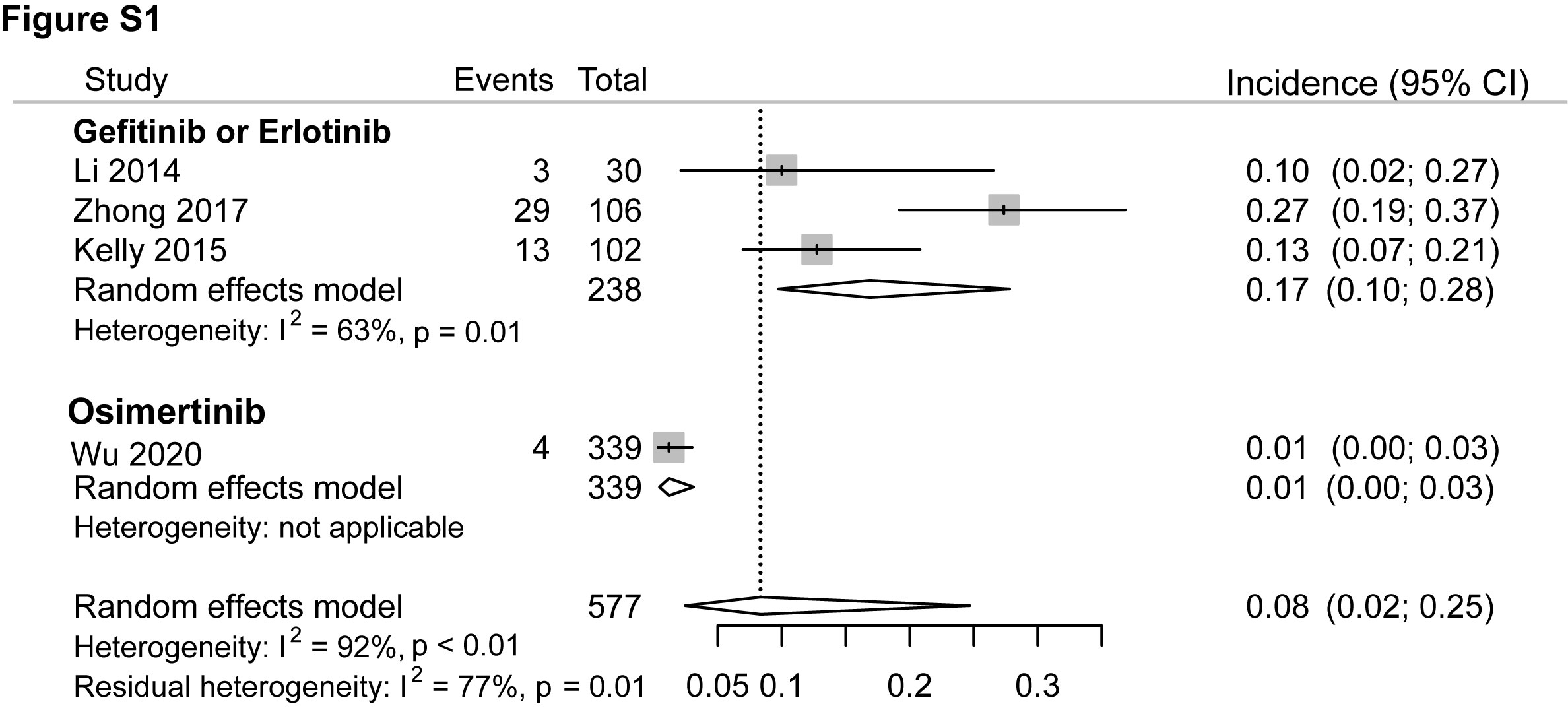

Supplement: Supplementary Figure 1 — Forest plots ot the incidence of brain recurrence. [file Image_1.jpeg]
